# Craniomandibular Disorders and Mandibular Reference Position in Orthodontic Treatment

**DOI:** 10.1155/2013/890942

**Published:** 2013-09-11

**Authors:** Farid Bourzgui, Hakima Aghoutan, Samir Diouny

**Affiliations:** ^1^Department of Orthodontics, Faculty of Dentistry, Hassan II University, Casablanca 20360, Morocco; ^2^Faculty of Letters & Human Sciences, Chouaib Doukkali University, El Jadida 24000, Morocco

## Abstract

The aim of this paper is to bring into focus the literature on the choice of the mandibular reference position in orthodontic treatment; of a particular reference to this paper is intercuspal position, centric relation position, or therapeutic position. To give a comprehensive account of the literature review on craniomandibular disorders (CMD), we have relied on books and articles using both Google Scholar and PubMed. Selection criteria included a combination of Mesh and type of article. Article classification was made by two authors, using the following structure outline: prevalence of craniomandibular disorders, its etiology and pathophysiology, occlusion and craniomandibular disorders, orthodontic treatment and CMD, and the mandibular reference position in orthodontics. An important conclusion that emerged from the present literature review is that CMD do not seem to be directly related to orthodontic treatment, and their appearance cannot be predicted or prevented by any means. Therefore, orthodontists must adopt a mandibular reference suitable to their patients and which best respects the balance existing in the stomatognathic system.

## 1. Introduction

Craniomandibular disorders (CMD) and their relevance to orthodontics have been a highly debated topic in recent years. Craniomandibular disorders (CMD) relate to discomfort of the temporomandibular joint (TMJ). The disorder is multifactorial with a degree of psychogenic influence varying throughout an individual's life with phases of symptoms affecting the quality of life. In an attempt to treat this complex group of disorders, several psychophysiological and psychological accounts have been proposed, but none of them was able to clearly elucidate a direct correlation with CMD (Michelotti and Iodice [1]).

CMD have been difficult to define. For example, Luther [2] used the term CDM to refer to the variety of symptoms, signs, and combinations that have been assigned to the TMJ and its related structures. Dibbets and van der Weele [3] commented that many different definitions of CDM dysfunction have come into existence and consequently, even in a single individual the diagnosis of TMJ dysfunction depends on the definition used. For the present study, the term “craniomandibular disorders” (or CMD) refers to a group of musculoskeletal conditions occurring in the temporomandibular region. These conditions are characterized by pain in the mastication muscles and in the TMJ, or both (Okeson and de Leeuw, 2011 [4]). These perturbations contribute to the deterioration of the quality of life of patients as well as their social functioning [5].

Traditionally it was believed that these disorders could be treated through gnathological occlusal principles. However, there are fundamental differences between gnathological and neuromuscular approaches in therapy when addressing the needs of patients who present with the numerous signs and symptoms that compromise the craniomandibular dysfunctional patient. Therefore, a new approach, referred to as the biopsychosocial model, came into existence; it is more scientific and widely accepted by the dental scientific community since its explanations rest on a medico-cognitive approach.

The aim of this study is to contribute to a better understanding of the nature of craniomandibular disorders by reviewing a number of books and articles. Specifically, it seeks to bring into focus the literature on the choice of the mandibular reference position (intercuspal position, centric relation position, or therapeutic position) based on clinical considerations and taking into account the balance of the stomatognathic system.

## 2. Materials and Method

For the present study, two search engines were employed to track down books and articles: Google Scholar and PubMed. Four books were classified and selected according to topic (Craniomandibular Disorders in Orthodontics). To track down articles, we first used “Craniomandibular Disorders” as major MeSH subheading including analysis, epidemiology, etiology, physiopathology, and therapy. The date of publication was not taken into account. This first step allowed us to have about 7308 articles; to restrict the number of articles, we used the Boolean to associate “Craniomandibular Disorders” to “orthodontics”. After this process, we got about 1667 articles. “Dental occlusion” as a key word was added to the previous two terms. This step allowed us to limit the number of articles to 214, which, in turn, was limited to 54 using the following selection criteria: clinical trial, meta-analysis, and review. 

## 3. Results

An examination of the review of the literature on craniomandibular disorders reveals that approximately 75% of the population have at least one sign of CMD (abnormal jaw movement, joint noises, tenderness on palpation, etc.) and approximately 33% have at least one symptom (facial pain, joint pain, etc.) [6]. According to Saghafi and Curl [7], an estimated 85 to 95% of the population will exhibit one or more symptoms of CMD in their lifetime with 5 to 6% of the population reporting clinically significant CMD related jaw pain [8]. CMD affect children, adolescents, and adults. Egermark-Eriksson et al. [9] found that CMD are present in 16–25% of children, 30% of adolescents, and 60% of adults. Various other studies found these abnormalities in children of varying ages [10–13]. An increase in CMD prevalence with increasing age has been found in children [14, 15]. A difference in CMD prevalence between boys and girls during adolescence has also been reported, where CMD prevalence is higher and the severity of signs and symptoms more pronounced in girls compared with boys [15, 16]. General health problems are also more frequently seen in adolescents with CMD compared with a control group [16]. Furthermore, adolescents with recurrent headaches have more symptoms and signs of CMD compared with those without headaches [17], and children and adolescents with CMD often have other painful conditions [16]. Although previous studies found the prevalence of symptoms and signs of CMD to be similar in men and women [18], later studies have reported a higher prevalence among women [8, 19, 20].

In our context, two studies about prevalence of DMC have been undertaken; the first study consisted of a sample of 142 students at the Dental School of Casablanca revealed that 52,8% of students have at less one sign of DMC and pain was present in 17,5% of the sample (Bourzgui et al. [21]). The second study included all patients receiving orthodontic treatment at the Dentofacial Orthopedic Unit of the Dental School of Casablanca, during the different stages of treatment and over a period of 4 months. Distribution of the sample by joint noise shows that 14% of cases reported recent joint noise; 12.3% reported antecedent noise. The joint noise lasted more than a month in 92.9% of the cases and less than a month in 7.1% of the cases. The pain was periorbital in 22.1% of the cases, auriculo-angular in 55.5%, perioral in 11.2%, and cervical in 11.2%. Pain was moderate in 71.54% of cases and severe in 28.4% (Bourzgui et al. [22]).

The etiology and pathophysiology of CMD are poorly understood; the fluctuation of symptoms with successive activation and remission periods makes their study difficult. If the multifactorial aspect of the disorder is no longer a subject of inquiry, the role of different factors in CMD is still unclear and is yet to be elucidated. Over the years, many classification schemes for CMD's factors have been offered. Among the classifications that are frequently used is the one by de Boever et al. [23]; it is distinguished as follows:

- predisposing factors that increase CMD risk: structural factors (occlusal patterns, loss of calibration, etc.), tissue quality, systemic diseases, age, facial typology, and bruxism;
- trigger factors: macrotrauma or microtrauma, bruxism, and articular tolerance ability excess;
- perpetuating factors: mostly neglected but usually dominated by behavioral, social, and emotional status, they tend to be more predominant.

According to Palla [24], the influence of behavioral factors is more important than the severity of symptoms. In their study, Manfredini et al. [25] have shown that pain-related disability is strongly associated with depression and somatization. Other neurobiological mechanisms such as interference with endogenous regulator of the pain system, genetic factors as well as the disruption of the adrenergic function of the autonomic nervous system have also been put forward as contributing factors in the pathogenesis of CMD (Monaco et al. [26]; Rinchuse and Kandasamy [27]).

In addition, the stomatognathic system is a complicated structure, and patients usually adapt to their existing vertical dimension of occlusion. When compensation capacity is exceeded, weak structures such as teeth, muscles, and joints yield and the disease manifests itself [6]. In the same way, Winocur et al. [28] conclude that hyper-functions related to par-functional habits such as bruxism or use of chewing gum contribute significantly to the onset of joint pain and noise. The same conclusion was reached by Conti et al. in 2003 who found a positive association between parafunction and CMD [29].

For several decades, the claim that occlusion plays a significant causal role in CMD has been debated and a substantial body of literature that investigates this issue has seen the light. The belief in this causal relationship was originally based on direct clinical observation. Recently, a number of researches have challenged this view, claiming that existing scientific literature “does not support” this hypothesis. Researchers such as Luther [2], John et al. [30], and Badel et al. [31] did not find any strong support for an occlusal etiology of CMD, at least not as a unique or dominant factor [6, 11, 32, 33]. Pullinger and Seligman [34] estimated that the contribution of occlusion to CMD is minimal and in most cases does not exceed 10–20%. They further suggested that the role of occlusion in TMD, quite apart from the issue of causation, may be more related to its potential as a perpetuating factor. However, Luther [2] and others (John et al. [30], Badel et al. [31]) argued that there is no causal relationship between occlusion and CMD. They further noted that because of flaws in investigatory design, the causative association between dental occlusion and TMJ has not been invalidated and remains an open question. Kirveskari and Alanen [32] have stated that “much, if not most, of the confusion about the role of occlusion is deeply rooted in a lack of appreciation of the problems in causal inference.”

In their study, McNamara et al. [35] claimed that the absence of an ideal gnathologic occlusion at the end of orthodontic treatment is not likely to lead to CMD. On the contrary, they classified five factors as statistically significant; they correlate perfectly well with their appearance: the previous skeletal open bite, the occlusal overbite exceeding 6–7 mm, the unilateral cross bite, the absence of more than five later teeth, and sliding between centric position and intercuspal position exceeding 4 mm. Marzooq et al. [36] found out that studies present conflicting scientific evidence in relation to the claim that malocclusions, such as overbite, passive interferences, and sliding between the occlusion of maximum intercuspidation and centric occlusion, contribute to CMD development. 

Today, there is good scientific evidence that the role of the occlusion should not be overrated to avoid surdiagnostics and overtreatment (Türp and Schindler [37]). It, therefore, should continue to be an important component of therapy practices and may constitute one of the main factors of development of the stomatognathic system.

The possible association between orthodontic, orthopedic, or orthochirurgical treatment and CMD has frequently been a subject of debate among clinicians in the last decades (Rtun et al. [38]; Beattie et al. [39]).

Despite the great number of studies, many doubts concerning the real participation of orthodontic treatment in the etiology, prevention, and treatment of CMD are still uncertain. Therefore, most researchers agree on the absence of causal relationship between orthodontics and CMD (Bourzgui et al. in 2009 [21]; Luther in 1998 [40]; Henrikson et al. in 2000 [41]; Conti et al. in 2003 [29]; McNamara et al. in 1995 [35]). In fact, a number of conditions (i.e., muscle incoordination, unstable disc-condyle relationship, and bone alterations) can interfere with the occlusal relationship and with orthodontic analysis. 

According to McNamara [42] CMD may develop during orthodontic treatment; there is no evidence that orthodontic mechanics can expose the subject to a higher risk for CMD, and there is little evidence that orthodontic treatment can prevent CMD. Furthermore, Conti et al. [43] showed that orthodontic treatment undertaken during adolescence can neither augment nor diminish the risk of developing CMD later. This is valid regardless of which mechanics is used: with or without extractions and with or without orthopaedic appliances. 

Al-Riyami et al. showed an improvement of articular noise (portray bangs rather than clicking) after orthognathic surgery. Also the limitation of oral opening and deduction seems to disappear two years after surgery [44]. This claim contradicts the findings of Borstlap et al. [45] who believe that orthognathic surgery can draw away effects, which are likely to contribute to CMD development. Luther et al. could not identify any single evidence regarding the preventive role of orthodontic treatment in CMD. The authors have also concluded that patients' consent should reflect the seemingly elusive character of episodic development/signs of reliefs [46]. 

## 4. Discussion 

Orthodontists should be able to handle such clinical situations, basing his work on scientific evidence and considering the multifactorial aspect of such trouble. They must also be able to distinguish patients with a risk and patients without a risk. During intervention, they must opt for criteria that favor occlusal stability while maintaining its functions [47]. In addition, orthodontic treatment is considered an occlusal therapy which should be done with mandibular reference position for occlusion reconstruction. The reference system assesses changes made relative to the initial state, but also to transfer information from the clinical to the laboratory and vice versa. But the question that poses itself is as follows: which reference to choose during orthodontic treatment especially in the presence of CMD? This issue has attracted considerable controversy.

The concept of reference implies a reproducible and recordable situation, which is not affected by the proposed treatment. Three possibilities are offered in this context (Orthlieb et al. [48]):

- intercuspal occlusal position (IOP);
- centric relation occlusal position (COP);
- “therapeutic mandibular position" is the position that you want the mandible to be treated with.

### 4.1. Intercuspal Occlusal Position (IOP)

This is the mandibular position that involves contact between the teeth while swallowing. In this position, there are an infinite number of condylar positions in the glenoid cavity.

### 4.2. Centric Relation (CR)

Centric relation is defined as the relationship of the mandible to the maxilla when the condyles are in their most posterior unstrained positions in the glenoid fossa [27, 49].

According to Türp et al. [50], the definition of centric relation has changed over the past half-century from a retruded, posterior and, for the most part, superior condyle position to an anterior-superior condyle position.

CR is used when restoring edentulous patients with removable or either implant-supported hybrid or fixed prostheses. Because the dentist wants to be able to reproducibly relate the patient's maxilla and mandible, but the patient does not have teeth with which to establish his or her own vertical dimension of occlusion, another method has been devised to achieve this goal. The condyle can only be in the same place as it was the last time it was positioned by the dentist if it is consistently moved to the most superior and anterior position within the fossa.

Centric relation believers [27, 49, 50] state the relationship of the mandible to the maxilla when the properly aligned condyle-disc assemblies are in the most superior position against the eminentiae irrespective of Occlusal Vertical Dimension (OVD) or tooth position. Centric relation concepts have largely been replaced by neuromuscular dentistry concepts that are considered far more physiologic. At the most superior position, the condyle-disc assemblies are braced medially, thus centric relation is also the mid-most position. A properly aligned condyle-disc assembly in centric relation can resist maximum loading by the elevator muscles with no sign of discomfort. The definition of centric relation may change with greater understanding of mandibular movement. Every individual has a position appropriate to him, and there is no single position of “normal” centric relation [27].

### 4.3. Which Treatment Method to Choose: CP or IOP?

A number of researchers such as Türp and Schindler [37] assume that the orthodontic approach is associated with a complete occlusal rehabilitation. Therefore, diagnosis and treatment can only be done by RC in order to achieve coordination between the occlusion and the masticating function showing whether the patient is symptomatic or not [51].

According to Oltramari et al. [52], centric relation (CR) is the position of the jaws in which the condyles have an orthopedic stable position. Thus, for any shift of centric position (CP) intercuspal occlusal position (IOP) causing changes in intercade sagittal relationship, diagnosis, and treatment should be based on the analysis in CR. IOP will only be used if it dictates the mandibular position by a maximum of stabilizing and harmoniously spreading contacts in a position close to centric relation without transversal differential.

However, in patients with CMD, the use of the CP is questionable, since it has been defined for an asymptomatic stomatognathic system [53]. However, Rinchuse and Kandasamy distinguish two approaches in orthodontic treatment [49], gnathological and nongnathological, and conclude that the condylar position in the fossa does not condition the appearance of CMD and articulator mounting as well as the determination to harmonize CR and IOP brings about very little or no benefit in orthodontics.

Hamata et al. [53] showed that there is no difference between the splints made in CP or IOP for patients with a good occlusal stability without large discrepancy between CP and IOP. To defend the IOP as a reference, a number of studies have shown that after a mandibular repositioning in the CP by successive adjustments of the splints, the final neuromuscular position of the mandible, which is asymptomatic, differs from the position at the beginning of treatment (CP) [53].

According to Tripodakis et al. [54], the neuromuscular position is located between IOP and CR in the antero-posterior direction. So the IOP position can be taken as a starting point for neuromuscular equilibrium position, because it is easier to perform and reduces the processing costs and the time spent in orthodontic treatment. 

To conclude, much controversy exists in the literature regarding the most reliable reference in orthodontics. But it is important to retain the simplified approach of Orthlieb et al. [48]. If an IOP is not affected by the treatment undertaken as a result of a mandibular repositioning which is itself resulting from a disk displacement redaction (DDR), it should be used. Any disruption of the IOP by a centering or sitting defect must choose the CR as a reference. In this case, it must be functional, that is, either natural or stabilized.

## 5. Conclusion

At the current state of research, CMD do not seem to be directly related to orthodontic treatment, and their appearance cannot be predicted or prevented by any means. Therefore, one needs to be vigilant in examining and approaching each patient before, during, and after orthodontic treatment, especially when risk factors dominate the clinical picture. So when the orthodontist is faced with the presence of signs, symptoms, or problems related to internal, articulatory disturbances, he should treat these disturbances before continuing treatment, especially that they can be the cause of morphological disorders in young patients. In this case, the noninvasive reversible means remain the most appropriate methods to use. In her/his treatment, the orthodontist must adopt a mandibular reference adapted to his patient and which best respects the balance existing in the stomatognathic system.
